# SARS-CoV-2 delta (B.1.617.2) spike protein adjuvanted with Alum-3M-052 enhances antibody production and neutralization ability

**DOI:** 10.3389/fpubh.2022.976686

**Published:** 2023-01-06

**Authors:** Hong Huang, Zhongcheng Zhou, Xinxin Xiong, Zhihai Liu, Xiaoxue Zheng, Qingli Quan, Meixing Yu

**Affiliations:** ^1^Guangzhou Women and Children's Medical Center, Guangzhou Medical University, Guangzhou, China; ^2^NHC Key Laboratory of Male Reproduction and Genetics, Guangdong Provincial Reproductive Science Institute (Guangdong Provincial Fertility Hospital), Guangzhou, China

**Keywords:** adjuvant, 3M-052, spike protein, antibody, SARS-CoV-2 (B.1.617.2)

## Abstract

**Background:**

Optimizing adjuvant is one of the critical methods to improve the vaccine. 3M-052, a novel TLR7/8 agonist which was designed for slow dissemination at the injection site, has a potential as adjuvant, but its performance as a vaccine adjuvant for SARS-CoV-2 (B.1.617.2) spike protein has not been studied. The present study aimed to evaluate the effect of Alum-3M-052 as an adjuvant to improve mice serum antibody titers and pseudovirus neutralization efficiency.

**Method:**

Female Balb/c mice were immunized 3 times at day 0, 7 and 21 intramuscularly with SARS-CoV-2 (B.1.617.2) spike protein and adjuvant (Alum or Alum-3M-052). Mice serum was collected weekly since day 7. Antibody titers of mice serum anti-SARS-CoV-2 (B.1.617.2) IgG and IgM were detected by ELISA. Inhibition rates of mice serum blocking SARS-CoV-2 (B.1.617.2) spike protein binding to ACE2 were detected by SARS-CoV-2 (B.1.617.2) Inhibitor Screening Kit. Neutralization efficiencies of mice serum against both SARS-CoV-2 (BA.2.12.1) pseudovirus and SARS-CoV-2 (B.1.617.2) pseudovirus were detected by pseudovirus neutralizing assay.

**Result:**

Serum of mice immunized by SARS-CoV-2 (B.1.617.2) spike protein adjuvanted with Alum-3M-052 had highest antibody titers and higher neutralization efficiency against both SARS-CoV-2 (BA.2.12.1) pseudovirus and SARS-CoV-2 (B.1.617.2) pseudovirus. Besides, neutralization efficiency of anti-SARS-CoV-2 (B.1.617.2) spike protein antibody against SARS-CoV-2 (BA.2.12.1) pseudovirus was lower than that of SARS-CoV-2 (B.1.617.2) pseudovirus.

**Conclusion:**

Alum-3M-052 rapidly increased the titer of anti-SARS-CoV-2 (B.1.617.2) spike protein neutralizing antibodies and enhanced the neutralization ability against pseudoviruses and variants. This study provided evidence for the application of Alum-3M-052 as an adjuvant in COVID-19 vaccines production.

## Introduction

After the first identification in Indian in December 2020, SARS-CoV-2 (B.1.617.2), one of Delta variants, spreads throughout about 175 countries rapidly (https://cov-lineages.org/global_report_B.1.617.2.html). Just 1 year later, the emergence of the Omicron once again refreshed scientists' understanding of SARS-CoV-2 transmission rate: past 1 million of new daily COVID-19 case at the end of 2021, according to Reuters. SARS-CoV-2 and its variants invade lung cells by binding to angiotensin-converting enzyme 2 (ACE2) *via* the receptor binding domain (RBD) of the virus spike protein (1). Antibodies aganist SARS-CoV-2 spike protein can effectively prevent the transmission of SARS-CoV-2 and its variants by inhibiting virus invading lung cells (2). Therefore, vaccines are one of the most effective ways to prevent the circulation of SARS-CoV-2 and its variants.

The Delta variants (B.1.617.2) has 23 mutations compared with the Alpha strains (3). New mutations enhanced the binding ability of the spike protein to the human ACE2, which thus makes the B.1.617.2 strain more contagious (3). The Delta variants were remarkably less sensitive to both serum neutralizing antibodies from recovered patients and vaccine-elicited antibodies, compared with the wild type bearing D614G (4). Despite the high vaccination rates and high prevalence of SARS-CoV-2 infection, the Delta variants still keep increasing virus replication fitness and reducing sensitivity to neutralizing antibodies (5). More troublesome is Omicron variant (especially BA.2.12.1). Liu et al. reported that mutations of Omicron variant at 452 (L452R and L452Q) of BA.2.12.1 might be one of the key drivers of neutralization resistance (6). As a result, similar to B.1.617.2, BA.2.12.1 variant can also evade immunity from the past infections and current vaccines (7). Hence, it is urgent matter to improve protective power of vaccines.

Adjuvants, as a critical component of vaccines, induce high titer and long-lasting antibody response (8). TLR agonists, one of the common vaccine adjuvants, recognize pathogens through pathogen-specific molecular patterns (PAMPs) to induce antimicrobial and inflammatory responses that affect innate and adaptive immunity (9, 10). It has been reported that TLRs agonists like imidazoquinolines imiquimod (R-837, TLR7 agonist) and resiquimod (R-848, TLR7/8 agonist) can active the TLR7/8 receptors on dendritic cell, and induce TH1 type of adaptive immune response (11). However, one of the limitations of resiquimod and other similar TLR7/8 agonists as vaccines is they are distributed rapidly throughout the body after injection, leading to systemic cytokines induction (12). 3M-052 is a novel TRL7/8 agonist. Due to its lipid-modified physical properties, 3M-052 stay at vaccination site and enhance the local TH1 immune response without inducing systemic cytokine production (13). Moreover, alum have been used clinically as adjuvant for more than half a century, which is believed to contributes to the uniform distribution of adsorbed antigens (14). Recently, Alum-3m-052 showed stronger virus neutralization ability in HIV-1 vaccination studies (9). Hence, we speculated that Alum-3M-052 has potential as an adjuvant.

Here, we used Alum-3M-052 as an adjuvant to immunize mice with SARS-CoV-2 (B.1.617.2) spike protein to evaluate the improvement of serum antibody titers and virus neutralization ability. We found Alum-3M-052 significantly enhanced antibody titers and higher neutralization efficiency against both SARS-CoV-2 (BA.2.12.1) pseudovirus and SARS-CoV-2 (B.1.617.2) pseudovirus.

Our research will provide data support for the clinical application of 3M-052 as a novel SARS-CoV-2 vaccine adjuvant.

## Materials and methods

### Mice vaccination

For each group, female Balb/c mice (6–8 weeks of age, *n* = 4) were immunized 3 times at day 0, 7 and 21 intramuscularly. Spike protein (Sino Biological, Beijing, China) was dissolved in PBS at the final concentration of 0.1 μg/μl. Alum-3M-052 was a mixture that contained 50 μl 2% aluminum hydroxide gel adjuvant (InvivoGen, France) and 2 μg 3M-052 (MedChemExpress, NJ, USA) in 50 ul PBS. For vaccine test group, 50 μl spike protein solution and 50 μl Alum-3M-052 were mixed at 150 rpm for 30 min by a shaker (Kylin-Bell, Jiangsu, China) at 4°C. For vaccine control group, 50 μl spike protein solution and 50 μl 2% aluminum hydroxide gel adjuvant were mixed at 150 rpm for 30 min by a shaker at 4°C. For negative control group, 50 μl PBS and 50 μl 2% aluminum hydroxide gel adjuvant were mixed at 150 rpm for 30 min by a shaker at 4°C. The blood samples were collected 1 week following each immunization.

### ELISA for anti-SARS-CoV-2 (B.1.617.2) IgG/IgM titer detection

ELISA plates (R&D, MN, USA) were coated with 2.5 μg/ml SARS-CoV-2 (B.1.617.2) spike protein (spike protein was diluted in PBS) and incubated at 4°C overnight. The plates were washed 3 times by wash buffer (R&D, MN, USA). Then, the plates were incubated with diluted serum samples for 1 *h* at 37°C and washed 3 times by wash buffer. The plates were incubated with diluted AP-conjugated goat Anti-mouse IgG (Yeasen, Shanghai, China) (1:500) for 1 *h* at 37°C. Then the plates were washed 3 times by wash buffer and developed with pNPP (Sigma, Darmstadt, Germany). Reactions were stopped with 3M NaOH (Sigma, Darmstadt, Germany). The optical density was determined at 450 nm. According to the above method, IgM was detected by AP-conjugated Goat Anti-mouse IgM (Abcam, Cambridge, UK) as the secondary antibody.

### Inhibition rates of mice serum blocking SARS-CoV-2 (B.1.617.2) spike protein binding to ACE2

We used the SARS-CoV-2 (B.1.617.2) Inhibitor Screening Kit (Spike RBD) (ACRO, Beijing, China) to detect inhibition rates of mice serum blocking SARS-CoV-2 (B.1.617.2) spike protein binding to ACE2. Positive control, negative control and 50-fold diluted mice serum were added to micro-plates coated with ACE2 protein, respectively. After 1 *h* incubation at 37°C, micro-plates were washed 3 times. Then, 100 μl of substrate solution was added in each well of micro-plates and the micro-plates were incubated at 37°C for 20 *min*. Finally, 50 μl of stop solution was added in each well and the absorbance (OD) was measured at 450 nm.


$$\begin{array}{l} \text{Inhibition}\;\text{rate = }({\text{OD}}_{\text{sample}}{\text{− OD}}_{\text{negative}\;\text{control}})\text{/ }\\ \;({\text{OD}}_{\text{positive}\;\text{control}}{\text{− OD}}_{\text{negative}\;\text{control}})\\ \;\times \text{100}\%. \end{array}$$

### Pseudovirus neutralizing assay

HEK-293T cells were seeded (5 × 10^6^) in a 100 mm dish and were co-transfected with 12 μg Plove-luciferase-EGFP plasmid, 6 μg psPAX2 plasmid and 2 μg spike protein variant (B.1.617.2 or BA.2.12.1) plasmid using Lipofectamine 3000 (Invitrogen, CA, USA) according to the manufacturer's instructions. After 8 *h* post-transfection, the medium was replaced with new culture medium. Two kinds of pseudotyped viruses were collected and filtered through 0.45 μm filter after 48 *h* transfection. RNA of pseudoviruses were extracted using MiniBEST Viral RNA/DNA Extraction Kit Ver.5.0 (TaKaRa, Otsu, Japan). Reverse transcription was conducted according to the protocol of HiScript^Ⓡ^ III All-in-one RT SuperMix Perfect kit (Vazyme, NJ, USA). RT-PCR was performed by TransLv Lentivirus qPCR Titration Kit (TransGen, Beijing, China).

To assess the neutralization of SARS-CoV-2 (B.1.617.2 or BA.2.12.1) infection, the 293T cells (1.2 × 10^4^/100 μL/well) were seeded in a flat-bottom culture 96-well plates and incubated overnight. Subsequent virus (~2 × 10^4^ RLU) was mix with 6 serial three-fold dilutions (50-folds as initial dilution) of mice serum in a 96-well plate. The mixture was incubated at 37°C for 1h and then transferred to the flat-bottom culture 96-well plates with seeded 293T cells. 6 virus control well (containing cells with virus) and 6 background control well (only containing cells) were also added in each 96-well plate. After 8 *h* incubation, the medium was replaced by fresh culture medium. Luciferase substrate was added to the 96-well plate (60 μL/well) after another 48 h incubation. The luminescence signal was detected by TECAN Infinite 5 min later.

### Statistical analysis

Statistical analysis was perform using the GraphPad Prism 8.0. Comparisons among three groups were performed with one-way ANOVA test followed by Tukey's multiple comparison test or Brown-Forsythe test. Comparisons between two groups were performed with unpaired Student's *t*-test. A *p*-value of <0.05 was considered statistically significant. ^*^p<0.05, ^**^p<0.01, ^***^p<0.001.

## Result

SARS-CoV-2 (B.1.617.2) with adjuvant Alum-3M-052 induced robust specific IgG antibody responses in mice.

**Figure 1:**
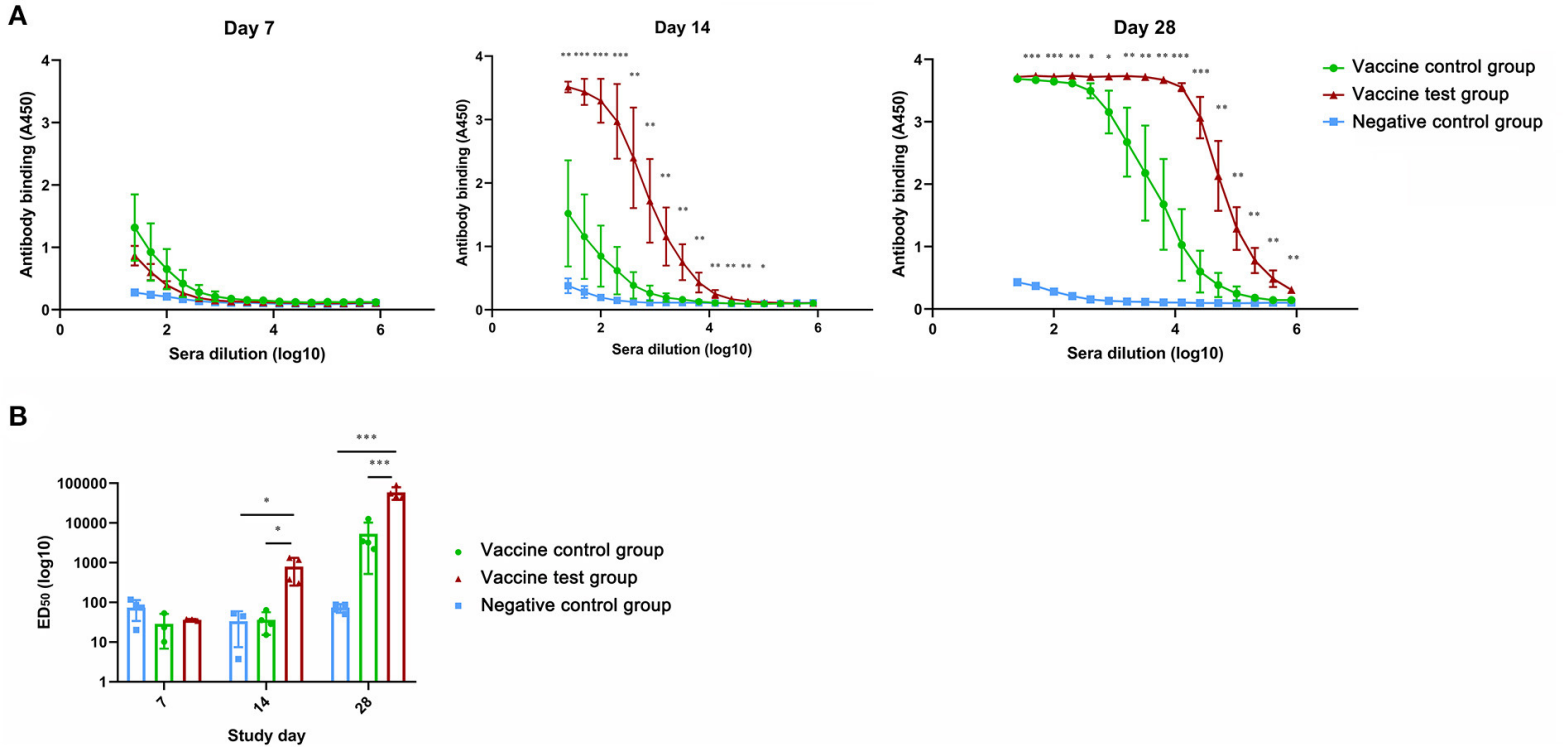
Anti-spike protein (B.1.617.2) IgG antibody titers in mice serum of three groups. **(A)** Anti-spike protein (B.1.617.2) IgG antibody titers in mice serum of three groups at day 7, 14, and 28. **(B)** Comparisons of ED_50_ titers of mice serum Anti-spike protein (B.1.617.2) IgG antibody in three groups at day 28 (Vaccine test group, *n* = 4; Vaccine control group, *n* = 4; Negative control group, *n* = 4; *p < 0.05; **p < 0.01; ***p < 0.001 *p* < 0.05 was considered statistically significant.).

We compared the ELISA results of three groups to evaluate the promoting effect of adjuvants in antibody production (Supplementary data 1). Absorbance values of serum (diluted from 25 to 204800 times) collected at day 14 and day 28 were significantly higher in vaccine test group compared with other two groups (Figure 1A). Absorbance values of serum in vaccine control group were increased in vaccine control group at day 28, which were remarkably higher than that in vaccine negative group (Figure 1A). Moreover, at day 14, the ED_50_ of IgG antibody of vaccine test group was significantly higher than that of vaccine control group (Figure 1B). At day 28, the ED_50_ of vaccine test group reached 59,055, which was remarkably higher than the other two groups (Figure 1B). ED_50_ was increased in vaccine control group at day 28, but there was no significant difference compared with vaccine negative group (Figure 1B). These results suggested that the adjuvant Alum-3M-052 increased IgG antibody productions.

### SARS-CoV-2 (B.1.617.2) with adjuvant Alum-3M-052 induced IgM antibody responses in mice

**Figure 2:**
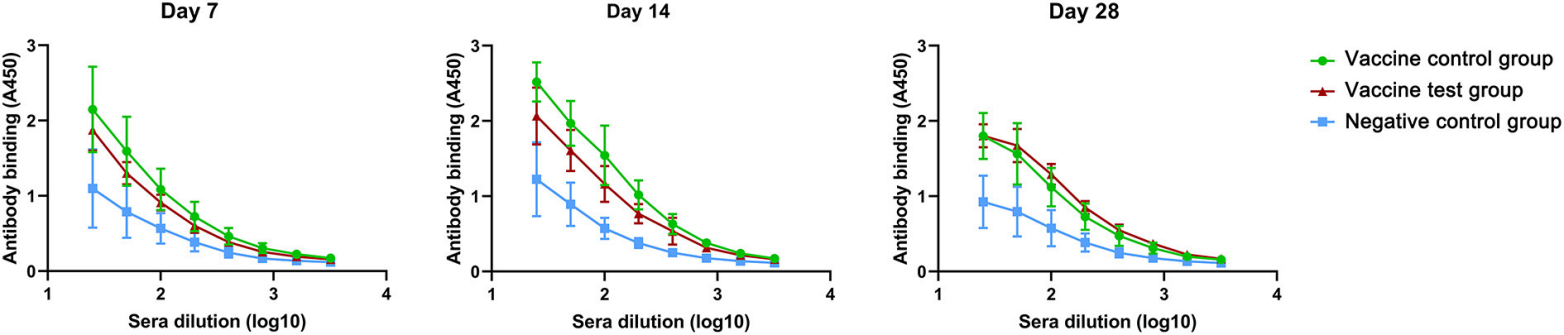
Anti-spike protein (B.1.617.2) IgM antibody titers in mice serum of three groups at day 7, 14, and 28 (Vaccine test group, *n* = 4; Vaccine control group, *n* = 4; Negative control group, *n* = 4.).

Next, we compared IgM antibodies titers in three groups (Supplementary data 2). The IgM absorbance values of the vaccine test group were lower than those of the vaccine control group in day 7 and day 14, but there was no statistical difference. The absorbance values of vaccine control group decreased in day 28 (Figure 2). In vaccine test group, the titers of IgM antibodies were more stable than those in vaccine control group.

### SARS-CoV-2 (B.1.617.2) with adjuvant Alum-3M-052 promoted neutralizing antibodies productions in mice

**Figure 3:**
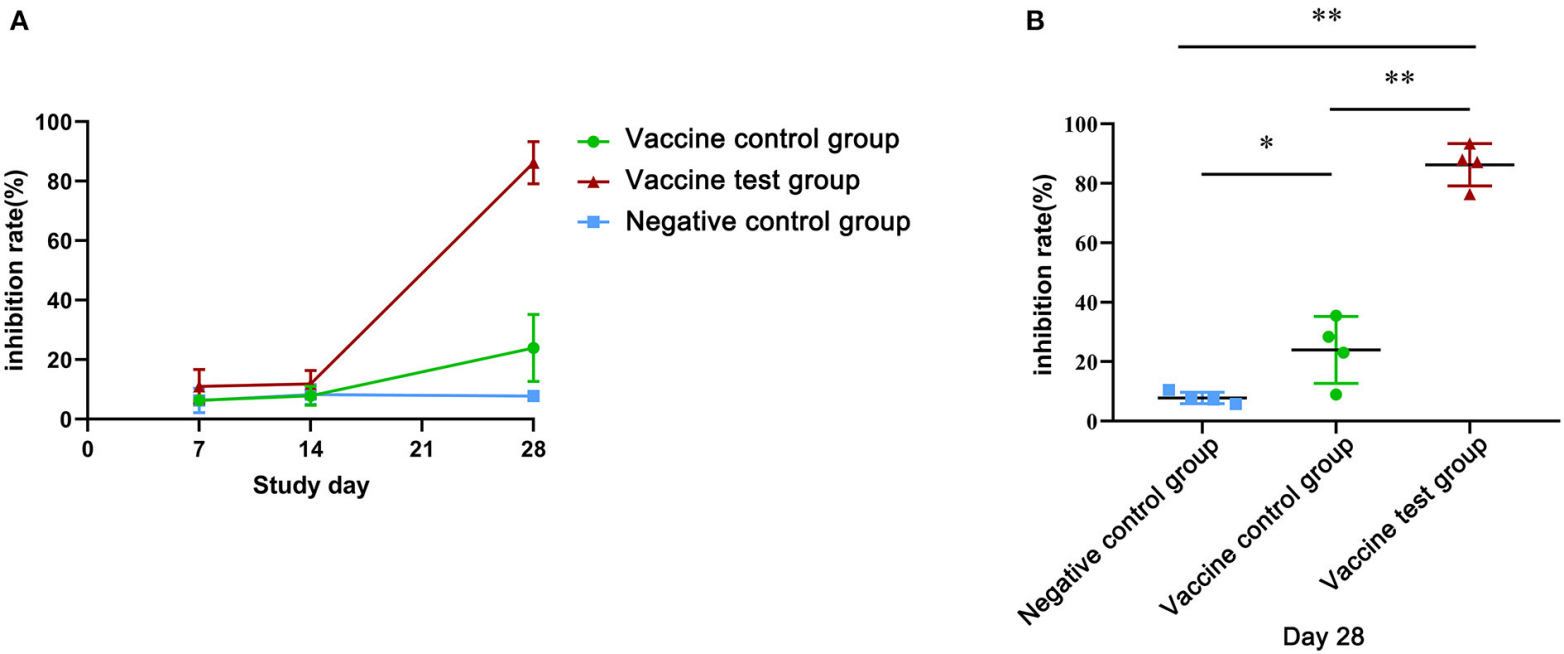
Inhibition rates of neutralizing antibodies blocked the SARS-CoV-2 (B.1.617.2) spike protein binding to ACE2. **(A)** Inhibition rates of mice serum neutralizing antibodies in three groups at day 7, 14, and 28. **(B)** The comparison of inhibition rates of neutralizing antibodies in the three groups on day 28 (Vaccine test group, *n* = 4; Vaccine control group, *n* = 4; Negative control group, *n* = 4; *p < 0.05; **p < 0.01; *p* < 0.05 was considered statistically significant.).

To further investigate effect of serum neutralizing antibodies on inhibiting the binding of the of SARS-CoV-2 (B.1.617.2) spike protein to ACE2, we performed an ACE2 competitive binding assay (Figure 3A) (Supplementary data 3). At day 28, the inhibition rate of serum antibodies in the vaccine test group was 86.22%, which was significantly higher than that of other two groups (Figure 3B). Additionally, in vaccine control group, the inhibition efficiency was 23.95%, which was significantly higher than that of the negative control group (Figure 3B). At day 7 and day 14, all the inhibition rates were low in three groups, and there was no significant statistical difference (Figure 3B). Therefore, Alum-3M-052 significantly improved the inhibition efficiency of serum neutralizing antibodies on the binding of SARS-CoV-2 (B.1.617.2) spike protein to ACE2.

### SARS-CoV-2 (B.1.617.2) with adjuvant Alum-3M-052 induce higher pseudovirus neutralizing antibodies in mice

**Figure 4:**
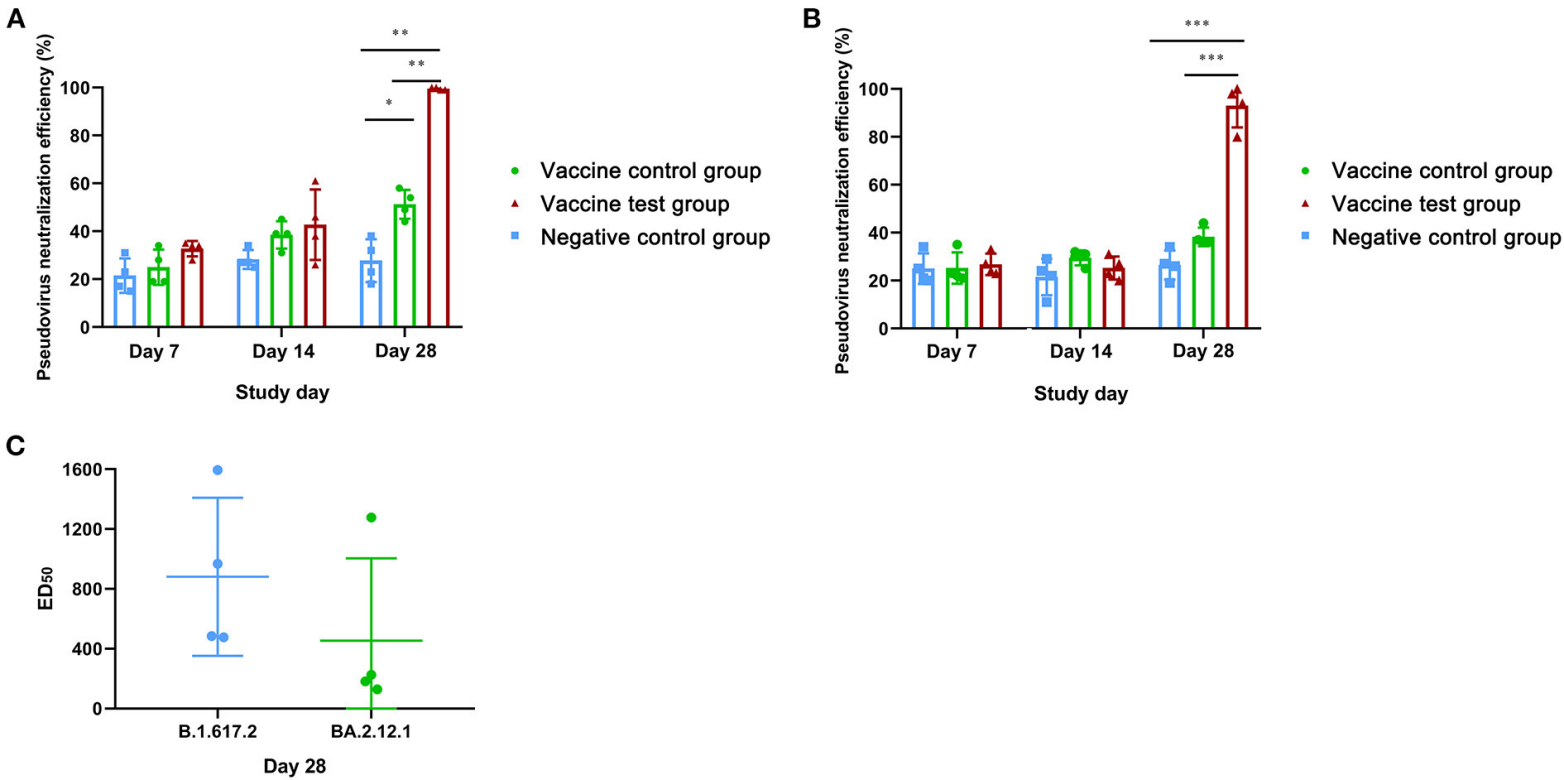
Neutralization efficiencies of mice serum neutralizing antibodies against pseudovirus of SARS-CoV-2 (B.1.617.2) and SARS-CoV-2 (BA.2.12.1). **(A)** Neutralization efficiencies of mice serum neutralizing antibodies against SARS-CoV-2 (B.1.617.2) pseudovirus in three groups at day 7, 14, and 28. **(B)** Neutralization efficiencies of mice serum neutralizing antibodies against SARS-CoV-2 (BA.2.12.1) pseudovirus at day 7, 14, and 28. **(C)** The comparison of ED_50_ of neutralization efficiencies between SARS-CoV-2 (B.1.617.2) pseudovirus and SARS-CoV-2 (BA.2.12.1) pseudovirus in vaccine test group on day 28 (Vaccine test group, *n* = 4; Vaccine control group, *n* = 4; Negative control group, *n* = 4; *p < 0.05; **p < 0.01; ***p < 0.001; p <0.05 was considered statistically significant.).

We also compared the pseudovirus neutralizing activity against SARS-CoV-2 (B.1.617.2) in three groups by pseudovirus neutralization assay for SARS-CoV-2 (B.1.617.2) (Supplementary data 4). At day 28, the neutralization efficiency of vaccine test group reached 99% at 50-fold dilution, which was significantly higher than the other two groups (Figure 4A). The neutralization efficiency of vaccine control group was significantly higher than that in negative control group (Figure 4A). At day 7 and day 14, there were no significant differences in neutralization efficiency among three groups (Figure 4A). Moreover, the mean ED_50_ of vaccine test group was 882 at day 28, while the mean ED_50_ of vaccine control group was less than 57 (Supplementary data 4). Results suggested that Alum-3M-052 improved the neutralizing efficiency against SARS-CoV-2 (B.1.617.2) pseudovirus.

Furthermore, we compared the neutralization efficiency of against SARS-CoV-2 (BA.2.12.1) pseudovirus in three groups. At day 28, the neutralization efficiency of vaccine test group reached 93% at 50-fold dilution, which was significantly higher compared with the other two groups (Figure 4B). Notably, the neutralization efficiency of vaccine control group was higher than the control group, but there was no statistical difference (Figure 4B). The mean ED_50_ of vaccine test group against SARS-CoV-2 (BA.2.12.1) subvariants was 454, which lower than that in SARS-CoV-2 (B.1.617.2) (Figure 4C). Results suggested that alum-3M-052 promoted the production of neutralizing antibodies against SARS-CoV-2 (B.1.617.2), and these neutralizing antibodies also had strong neutralizing efficiency against SARS-CoV-2 (BA.2.12.1).

## Discussion

Vaccine adjuvant works as one of the critical components of vaccines and induces high titer and long-lasting antibody response (8, 9). Small molecule TLR7/8-specific agonists have demonstrated potential as adjuvants, since they activate DCs and monocytes and thus enhance both humoral and cellular immune response (15, 16). Compared with resiquimod and imiquimod that distributed rapidly throughout the body after injection, 3M-052, a novel lipid-modified TRL7/8 agonist, stay at vaccination site and improve the potency of neutralizing antibodies (9). It was reported that Alum-3M-052 induces durable HIV-1 envelope-specific plasma cells and humoral immunity in nonhuman primate (10). What is more, Alum-3M-052 significantly improve titers of anti-SARS-CoV-2 RBD trimer protein antibody and thus prevent SARS-CoV-2 from infecting the lungs (17). Here, we immunized mice with SARS-CoV-2 (B.1.617.2) spike protein adjuvanted with Alum-3M-052. We found that Alum-3M-052 not only significantly increased the titers of anti-SARS-CoV-2 (B.1.617.2) spike protein neutralizing antibody in mice serum, but also dramatically improved the neutralization ability of serum antibodies to SARS-CoV-2 (B.1.617.2) pseudovirus and SARS-CoV-2 (BA.2.12.1) pseudovirus.

Serum neutralizing antibodies block the entry of SARS-CoV-2 virus into lung cells (7). In our study, Alum-3M-052 rapidly increased titers of anti-SARS-CoV-2 (B.1.617.2) spike protein antibody in mice serum and significantly increased the neutralization ability against SARS-CoV-2 (B.1.617.2) pseudovirus. The binding of the spike protein with host ACE2 receptor is the initial step of SARS-CoV-2 virus entry, and inhibition of this step is critical for preventing virus invasion (1). We noticed that neutralizing antibody titers in mice serum of the vaccine test group increased significantly at day7 after the second boost, but the neutralizing antibody was not enough to inhibit the binding of SARS-CoV-2 (B.1.617.2) spike protein to ACE2 protein, nor to prevent pseudoviruses invade target cells. Results indicated that not only antibody titer but also its binding quality is important for protection, especially when SARS-CoV-2 variants, which present higher affinity for ACE2. This partly explained why recovered patients and vaccinated patients are also at risk of coronavirus reinfection.

SARS-CoV-2 (BA.2.12.1) have exhibited poor susceptibility to neutralizing antibodies in sera of recovered patients and vaccinated populations (18). There are differences in protective efficacies of the SARS-CoV-2 vaccines against different mutant strains (19, 20). We found relatively high antibody titers provide effective protection against mutant strains in different degrees of range. We found that at day 7 after the third boost with SARS-CoV-2 (B.1.617.2) spike protein, neutralizing ability of mice serum against the SARS-CoV-2 (BA.2.12.1) pseudovirus was strong. This suggested that Alum-3M-052, as a vaccine adjuvant, has a potential to improve vaccine protection against new mutant variants.

Besides, based on the fact that higher IgG antibody titers and more stable IgM antibody titers in vaccine test group, we speculated Alum-3M-052 may improve antigen presentation of maturation of the immune response. This need to be confirmed in the further study. We will investigate the persistence, safety and immune mechanism of Alum-3M-052.

In conclusion, Alum-3M-052 not only rapidly increased the titer of anti-SARS-CoV-2 (B.1.617.2) spike protein neutralizing antibodies, but also enhanced the neutralization ability against pseudoviruses and variants. Our study provided a basis for the application of 3M-052 as an adjuvant in COVID-19 vaccine development.

## Data availability statement

The original contributions presented in the study are included in the article/Supplementary material, further inquiries can be directed to the corresponding author/s.

## Ethics statement

The animal study was reviewed and approved by Guangzhou Medical University Animal Care and Use Committee.

## Author contributions

MY and QQ designed the research and revised the language of the paper. HH and ZZ analyzed the data and wrote the draft. XX, ZL, and XZ collected the research data. All authors read and approved the final manuscript.

## Supplementary material

Supplementary data 1 Data of mice serum IgG antibody titers against spike protein (B.1.617.2).

Supplementary data 2 Data of mice serum IgM antibody titers against spike protein (B.1.617.2).

Supplementary data 3 Data of inhibition rates of mice serum neutralizing antibody against SARS-CoV-2 spike protein (B.1.617.2).

Supplementary data 4 Data of neutralization efficiencies of mice serum neutralizing antibodies against pseudovirus of SARS-CoV-2 (B.1.617.2) and SARS-CoV-2 (BA.2.12.1).
